# Can Sri Lankan Australians Recognise Depression? The Influence of Acculturation, Age and Experiences with Depression on Recognition

**DOI:** 10.3390/ijerph192214839

**Published:** 2022-11-11

**Authors:** Amanda Daluwatta, Dushan Peiris, Kathryn Fletcher, Chris Ludlow, Greg Murray

**Affiliations:** 1 Centre for Mental Health, School of Health Sciences, Swinburne University of Technology, Melbourne, VIC 3122, Australia; 2 Department of Psychological Sciences, School of Health Sciences, Swinburne University of Technology, Melbourne, VIC 3122, Australia

**Keywords:** mental health literacy, Sri Lanka, Australia, depression, problem recognition, help-seeking

## Abstract

Mental health literacy is an important determinant of mental health help-seeking and is associated with improved mental health. There is evidence that mental health literacy may be lower amongst some migrant communities in Australia. The present study conducted the first cross-sectional survey of mental health literacy in Sri Lankan Australians between April and October 2020. Participants (*N* = 404) were presented with a culturally-tailored vignette describing an individual with symptoms of major depressive disorder, with correct recognition determined by the coding of an open text response to the question ‘what’s wrong with Mr Silva?’. Binomial linear regression modelling was conducted to identify predictors of the correct recognition of depression. Approximately 74% of participants recognised the presented symptoms as depression, though multiple other labels were also used by the respondents. The results also suggested that younger age and having a prior diagnosis of depression were significant predictors of recognising depression in the vignette. In the first study of Sri Lankan migrants’ mental health literacy in an Australian context, the rates of depression recognition were comparable to those found in the general Australian population. Further research is urgently required to replicate and extend the present findings and ultimately support the development of tailored interventions aimed at improving mental health literacy across the diverse Sri Lankan Australian community.

## 1. Background

Asian migrants and refugees within Australia are less likely to use and report greater difficulty in accessing mental health services [1,2,3], despite a need for services being present [2]. In fact, Australian census data confirms that Asian-born individuals access subsidised mental health services less frequently than Australian-born residents (4.4% versus 7.8%) [4]. Investigations into the factors contributing to lack of service use for each specific ethnic group are limited, though necessary, in order to improve the ability of services to meet their mental health needs. Sri Lankan-born Australians are the 10th largest group of overseas-born residents [5], therefore representing a significant minority group in present day Australia [6]. In light of the high rate of suicide in Sri Lankan diasporic communities [7] and the psychological impact of the Civil War [7], Easter Sunday bombings [8,9], tsunami [10,11] and economic and political crises [12,13] on this population, the paucity of the literature relating to their experiences of mental illness and factors that shape their help-seeking behaviours is a key research priority.

One of the putative determinants of professional-help-seeking behaviour is better mental health literacy (MHL) [14,15]. Jorm et al. [16] defines MHL as “knowledge and beliefs about mental disorders which aid their recognition, management or prevention”. MHL can be divided into five components, including knowledge of prevention and risk factors of mental conditions; the ability to recognise specific conditions (typically clinical depression); knowledge of help-seeking options and treatments; knowledge of effective self-help strategies; and mental health first aid skills to support others who are experiencing a mental condition or a mental health crisis [17]. The current paper focused on Sri Lankan Australians’ ability to recognise depression rather than the other components of MHL, as it is postulated that being able to provide a psychiatric label can activate a schema about appropriate action that the individual could engage in [17].

No studies to date have investigated Sri Lanka Australians’ ability to recognise depression. However, numerous studies have investigated the recognition of depression in adults residing in Sri Lanka [18,19]. Amarasuriya et al. [18] found that 17.4% of Sri Lankan undergraduate students identified depression, and that their ability to accurately use psychiatric labels when presented with a vignette was the strongest predictor of their intentions to seek informal and professional help. Recognising the problem as depression was also associated with an endorsement of more professional help and treatment options [18]. The authors concluded that educating Sri Lankan undergraduate students to recognise psychiatric symptoms could trigger treatment beliefs in line with recommended help-seeking behaviours [18].

One’s ability to recognise depression may also vary as a result of individual differences [20]. For instance, studies in Australia have consistently demonstrated that males are less likely to correctly identify mental conditions (including depression) than females [21,22], and that younger individuals are more likely to identify the symptoms of depression than older individuals [23,24]. Furthermore, having a close friend or family member with a mental disorder has been found to be significantly associated with correct recognition of depression in Australia [24] and Singapore [25]. Additionally, respondents with higher education have exhibited greater ability to recognise depression in residents of Tehran city and suburban areas [26] as well as in Sri Lankan [27] and Australian populations [24]. Research has also generally found that East Asian immigrants are less likely to recognise symptoms of depression and schizophrenia compared to the general public [28].

Exploration of factors influencing MHL in specific cultural groups remains in its infancy [20]. There is some evidence that individual differences in acculturation (broadly defined as a two-way process of cultural and psychological change due to long-term contact between two or more cultural groups and their individual members [29]) predicts problem recognition among East Asian immigrants [28]. For example, utilising the original MHL vignette methodology, gender was found to be a predictor of correct identification of depression, with more female than male Chinese-speaking Australians correctly identifying depression [22]. However, the female participants had been living in Australia for longer and were more acculturated than their male counterparts, which may have accounted for the difference [22]. Studies that used questionnaire methodologies have also demonstrated that acculturation influences depression knowledge and recognition. For instance, Korean American elders with lower levels of acculturation were found to have less knowledge of depression [30]. Similarly, Parker et al. [31] found that Chinese Australians who had lower acculturation were more likely to recognise somatic symptoms of depression and less likely to recognise cognitive symptoms than participants with higher acculturation. However, Parker et al. [31] also found that acculturation was a less important predictor of recognition than prior personal experience of depression. In sum, much remains unknown about the predictors of depression recognition.

## 2. The Present Study

To the best of our knowledge, no previous study has assessed MHL amongst the general Sri Lankan Australian population. The present study sought to advance the understandings of MHL of Sri Lankan adults residing in Australia and had two aims. First, we aimed to characterise the MHL of this population in terms of participants’ ability to correctly identify depression as presented in a vignette. Second, we investigated a series of demographic and other predictors of correct recognition. It was that hypothesised greater acculturation, younger age and prior diagnosis of depression (Hypotheses 1, 2 and 3, respectively) would be associated with the correct identification of depression.

## 3. Methods

### 3.1. Design, Participants and Setting

Data was collected through an online cross-sectional survey between April 2020 and October 2020. Individuals were eligible to participate if they were at least 18 years of age, English-speaking, self-identified as being of Sri Lankan heritage and currently living in Australia.

### 3.2. Survey Development

After obtaining permission from the vignette’s original author, Professor Anthony Jorm, the survey underwent several stages of adaptation including incorporation of items relevant to the migrant experience and the Sri Lankan Australian culture. The survey was piloted with nine Sri Lankan Australians to assess cultural relevance and whether the questions were clearly written and received appropriate answers (face validity). Feedback was used to modify some scales in the survey (content validity).

### 3.3. Measures

#### 3.3.1. Demographics

The survey commenced with a series of demographic questions that assessed age, gender, education, country of birth, ethnicity, religion, number of years in Australia, age at migration, reason for migration and state/territory of current residence. Due to the concern of the confounding impact of COVID-19 during data collection [32], the survey also included a single item measuring the impact of the COVID-19 pandemic on the participant’s mental health and wellbeing (response options: 1—not at all affected, 2, 3, 4, 5—greatly affected).

#### 3.3.2. Personal Experience with Depression

In line with the literature [24], a series of items related to mental health history including if participants had previously received a diagnosis of depression from a health professional (response options: diagnosis, no diagnosis) and participant’s exposure to individuals with depression was presented. Participant’s exposure to depression was examined by asking if any of their family members or close friends had received a diagnosis of depression (response options: yes, no, don’t know) and if participant’s employment ever involved providing treatment or services to a person with depression (response options: yes, no).

#### 3.3.3. Acculturation

Acculturation was measured on a modified version of the commonly used Suinn-Lew Asian Self-Identity Acculturation (SL-ASIA) Scale. Although the original SL-ASIA comprises of 21 multiple-choice items [33], researchers have historically adapted the scale by altering the item wording, as well as by adding and removing items [34,35,36]. For example, Leong and Chou [34] created a five-item brief form of the SL-ASIA, which had a Cronbach’s alpha of 0.80 and was found to be highly correlated to the full-scale SL-ASIA (r = 0.91). In line with Parker et al.’s [37] adaption for the Chinese Australian population, the SL-ASIA was modified for use with Sri Lankan Australians, with the final scale including 12 items. Participants rated each item on a 5-point Likert scale, with a response of 1 indicating a low level of acculturation and a response of 5 indicating a high level of acculturation [33]. In line with the scoring procedure identified by Suinn et al. [33], a final mean acculturation score was calculated by summing the values of all the items and dividing the sum by 12. Each participant was given a final acculturation score ranging from 1 (Sri Lankan identified) to 5 (Australian identified). Validity results for the 12-item measure have not been published, but in the current study, a reliability analysis of the acculturation scale suggested that the internal consistency was excellent (α = 0.90).

#### 3.3.4. Recognition of Depression

Following previous investigations of problem recognition [18,38], this facet of MHL was measured on a culturally tailored vignette. The vignette described Mr. Silva, a 30-year-old Sri Lankan Australian with major depressive disorder consistent with DSM-5 diagnostic criteria. The below vignette was presented to participants:

*Mr. Silva has been feeling unusually sad and miserable for the last few weeks. Even though Mr. Silva feels tired all the time, Mr. Silva has difficulty falling asleep almost every night. Mr. Silva doesn’t feel like eating and has lost weight. Mr. Silva finds it difficult to concentrate at work and make decisions about day-to-day tasks. This has come to the attention of his boss who is concerned about his lowered productivity. Mr. Silva doesn’t want to socialize with family and friends anymore, and prefers to stay alone all the time. Mr. Silva seems very different to what Mr. Silva was like before. Mr. Silva’s wife and friends are very worried about Mr. Silva.*

The vignette was followed by an open-ended question, which asked, “What do you think is wrong with Mr. Silva?” (recognition of depression), and additional vignette-related MHL questions (not examined in the present paper).

### 3.4. Procedure

Ethical approval was obtained from Swinburne University Human Research Ethics Committee (20202610-4168). A purposive sampling strategy was used to recruit Sri Lankan Australians, and the online survey was distributed in English through social media accounts, university cultural groups and via community groups’ websites and radio. Participants were informed that their identity would be anonymous, and that participation was voluntary. Before participants commenced the questionnaire, they were invited to click ‘next’ indicating their consent to participate in the study and for their data to be analysed.

### 3.5. Coding of Responses for the Recognition of Depression Question

To facilitate comparisons with earlier studies [16,18,39], A.D. recorded responses according to response categories used in the above studies, where the correct response was any answer that contained a variant of the term “depression”. As similar work had not assessed the current population of interest before, new coding categories were created for responses that did not fit into the pre-coded categories. As part of the development of additional coding categories, a second operationalisation of correct recognition was also developed. In this second category, participants’ response was deemed correct only if they (a) mentioned the word ‘depression’ and (b) did not mention any alternative psychological or psychosocial issues in their text response. The aim of this innovative and narrower category was to characterise whether a given response specifically and unambiguously identified depression (potentially indicating a clearer opinion of the problem and forming a stronger basis for treatment seeking). This second operationalisation of correct recognition (labelled below as depression unambiguously recognised) was a subset of the category most commonly reported in the literature (depression recognised). Each of the categories obtained for this question were coded as ‘yes’ or ‘no’ so that multiple responses were possible [40]. For simplicity, the response categories reported here included those nominated by more than 1% of the sample.

In order to establish inter-rater reliability, a random sample of 45 responses (approximately 10% of responses) was independently scored by A.D. and D.P. There was almost perfect agreement between the raters on the following categories: depression (0.95), adjustment (1.0), anxiety (1.0), physical conditions (1.0), stress (1.0), mental problem (0.81), lack of motivation (1.0), social withdrawal and isolation (1.0) and other (1.0). Cohen’s Kappa was good for the life problems (0.65) category and fair for the mental illness (0.38) category. Kappa could not be calculated because of zero frequencies from one of the raters for the categories of lack of communication, trauma, emotional symptoms, don’t know and burnout. To better understand the reasons for the fair agreement on the mental illness category, positive and negative agreement were examined separately [41]. There was a 93.3% percent agreement between the raters for the mental illness category. As the inter-rater reliability estimates were high, A.D. proceeded to score the other responses of the sample, consulting with D.P. and G.M. whenever necessary.

### 3.6. Statistical Analyses

Statistical analyses were performed using IBM SPSS Statistics Version 27. The years in Australia variable was recoded into four categories and the ages of the participants were recoded into six categories. Furthermore, the sub-categories relevant to the ethnicity, country of birth and religion variables, which were of low occurrence, were recoded into ‘other’ categories.

Descriptive analysis was performed to assess the sociodemographic characteristics of the study participants (*N* = 404). For Aim 1, recognition percentages were presented as valid percent frequencies with 95% confidence intervals. A sequential binary logistic regression model was used to examine the predictors of the depression recognised variable (Aim 2). For consistency with the literature, only the traditional code (depression recognised) was investigated in Aim 2. Bivariate analyses (*t*-test or chi-squared, as relevant) were conducted to identify which demographic and other predictors should be entered into the multivariate model. Note, the three hypothesised predictors were to be included in sequential order whether or not they met bivariate significance. An assumption check was performed suggesting all continuous independent variables were linearly related to the logit of the dependent variable and that the data met the multicollinearity assumption [42]. Five standardised residuals with values greater than 2.5 standard deviations were also detected and kept in the analysis.

## 4. Results

### 4.1. Descriptive Findings and Sample Characteristics

A missing-values analysis found that there was progressive attrition from the survey, specifically, 87.5% of variables were complete, and only 4.4% of all values were missing. Due to the ordering of the items in the survey, only a subset (*n* = 262) of those consenting to participate (*N* = 404) provided data for the analysis of Aim 2.

Of the *N* = 404 adults (120 males, 284 females) who consented to participate, 84.9% identified as Sinhalese, 6.7% as Tamil and 8.4% belonged to another ethnicity (see Table 1). The mean age was 31.6 years (*SD* = 11.6 years; *range* = 18–77 years), and the mean final acculturation score was 3.0 (*SD* = 0.7; *range* = 1.6–4.6). Furthermore, 60.8% of participants indicated that their mental health and wellbeing were moderately to greatly affected by COVID-19. The demographic distribution of the smaller sample used in Aim 2 was not significantly different to the full sample described in Table 1. Of the *n* = 262 Sri Lankan Australians who completed the entire survey, 53.8% reported previous exposure to depressive symptoms through their family or close friends, and 32.4% reported previous exposure through their employment. Additionally, just over 30% of participants reported having a prior diagnosis of depression.

### 4.2. Recognition of Depression

Table 2 displays the final coding categories: depression recognised (correct recognition), depression unambiguously recognised, anxiety/worry, stress, mental problem, mental illness, physical condition, emotional symptoms, don’t know, has a problem (neither physical nor mental), social withdrawal and isolation, burnout, trauma/loss, lack of motivation, adjustment, lack of communication and other.

The finding for Aim 1 is shown in the first row of Table 2: 73.8% of participants correctly recognised the syndrome of depression in the vignette. When using the novel, more restrictive definition of depression recognition (i.e., depression unambiguously recognised), just over half of the participants (53.7%) correctly recognised the problem as depression. Following ‘depression recognised’, ‘anxiety’ and ‘stress’ were the most common categories nominated. In all, the majority of participants mentioned a category that could be regarded as being within the sphere of mental health.

### 4.3. Predictors of Recognition of Depression

First, bivariate analyses of predictor variables and the dependent variable were conducted to inform the regression model. The following predictors were entered in the multivariate model as they had bivariate associations with the dependent variable: prior diagnosis of depression *X^2^* (1, *N* = 262) = 7.58, *p* = 0.01; highest level of education *X^2^* (3, *N* = 262) = 24.04, *p* < 0.001; age t(260) = 6.77, *p* < 0.001; and acculturation t(260) = −3.98, *p* < 0.001. However, country of birth *X^2^* (2, *N* = 262) = 4.35, *p* = 0.11; gender *X*^2^ (1, *N* = 262) = 0.03, *p* = 0.88; ethnicity *X^2^* (2, *N* = 262) = 0.14, *p* = 0.93; religion *X^2^* (4, *N* = 262) = 5.54, *p* = 0.24; having a family member or close friend with depression *X^2^* (2, *N* = 262) = 3.69, *p* = 0.16; exposure to individuals with depression through employment *X^2^* (1, *N* = 262) = 2.52, *p* = 0.11; years in Australia t(260) = 1.17, *p* = 0.24; and impact of COVID-19 pandemic on the participant’s mental health and wellbeing *X^2^* (4, *N* = 262) = 3.07, *p* = 0.55 did not have significant relationships with the participants’ ability to recognise depression.

A four-stage sequential binomial logistic regression was performed to assess the prediction of a respondent’s ability to recognise depression, first on the basis of the hypothesised predictors and then after the addition of other demographic predictors (see Table 3). Model 1 examined the unique contribution of acculturation in a participant’s ability to recognise depression (H1). In Model 1, acculturation emerged as a statistically significant predictor of **a** participant’s ability to recognise depression. In Model 2, the acculturation variable was retained, and age was added as a predictor (H2). In this model, age was the sole significant predictor, and acculturation fell to non-significance. Participant’s prior diagnosis of depression was entered in Model 3 (H3). In Model 3, both age and prior diagnosis of depression emerged as significant predictors of recognising depression.

Model 4 contained four independent predictors (age, acculturation, highest level of education and prior diagnosis of depression) that had significant bivariate relationships with the dependent variable. Model 4 was statistically significant, χ^2^(6) = 50.44, *p* < 0.001. Sensitivity was 94.5%, specificity was 33.9% and the area under the ROC curve was 0.78, 95% CI [0.72, 0.85]. Of the four predictor variables, only three were statistically significant. Specifically, correct recognition of depression varied according to age, highest level of education attained, and prior diagnosis of depression. Participants with a diagnosis of depression were 2.93 times more likely than participants without a diagnosis to recognise depression in the vignette. Increasing age was also associated with a reduction in the likelihood of recognising depression. Additionally, the participants who had completed a TAFE course or equivalent as their highest level of education had 0.12 lower odds of recognising depression than participants who had completed secondary college as their highest level of education. After controlling for the other explanatory variables, acculturation was no longer a significant predictor.

## 5. Discussion

Over the past two decades, MHL has been promoted as a public health intervention that raises awareness, increases the well-being of the general population and promotes positive attitudes towards mental health and help-seeking [28]. The present study is the first to explore the nature of and predictors of recognition of depression (a core component of MHL) among the Sri Lankan Australian community. The correct identification of depression in the tailored vignette was 73.8%. The predictors of recognition of depression (Aim 2) were partly as expected; younger age (Hypothesis 2) and self-reported personal diagnosis of depression (Hypothesis 3) arose as significant independent predictors of correct identification, whereas acculturation level (Hypothesis 1) did not. These findings provide important information that can be used to inform targeted public health interventions for this population.

In the 2011 National Survey of Mental Health Literacy and Stigma, approximately 75% of the Australian adults surveyed were able to correctly recognise depression in a vignette [40]. These results are comparable to the current study that found that nearly 74% of Sri Lankan Australians identified depression in the vignette.

The relatively high rates of depression recognition found here contrast strongly with the Amarasuriya et al. [18] study of 4671 undergraduates in Sri Lanka, in which only 17.4% of the respondents recognised depression. This finding may be related to the majority of the current sample either identifying as bi-cultural or Australian (as suggested by the acculturation measure). Therefore, a direct comparison between the present study and that of Amarasuriya et al. [18] could not be drawn, and the cultural comparability of findings with samples in the country of origin were limited. Interestingly, a study by Wong et al. [15] also found that Chinese Australian participants had better recognition of depression (14.4%) than Chinese participants residing in Shanghai (12.1%) and Hong Kong (13.9%).

The depression recognition rate in the current study was also markedly higher than the rates identified in studies of other Asian migrant groups in Australia. In a sample of Chinese immigrants in Australia (age *M* = 44.7 years), 51% of respondents correctly labelled depression [43]. Another study among Chinese-speaking Australians found that only 14% of the respondents were able to correctly label the depression vignette [3]. It is likely that the higher rates of recognition in the current study compared to the Chinese Australian samples was related to our sample being significantly younger (age *M* = 31.6 years, versus *M* = 49.2 years in the study by Wong et al. [3]). Additionally, societal changes may shed light on our findings. For example, our sample had lived in Australia for an average of more than 17 years, and during that time (and since the Chinese Australian studies were conducted) many community interventions to improve MHL have been implemented in Australia [44]. Thus, the improved recognition rates for depression may partially reflect the efforts of Beyond Blue (an organisation that has been continuously promoting national depression initiatives in Australia since 2000), Headspace, SANE, the Black Dog Institute and Everymind [44].

While correct recognition appeared to be common in the present study, the results using our novel, restrictive definition of depression recognition (i.e., depression unambiguously recognised) indicated that approximately half of the participants were unable to specifically and unambiguously identify depression in the vignette, signifying that 20% of respondents could not discriminate depression from other psychological and psychosocial issues. In reality, this could potentially result in the misclassification of depression, and subsequently delay help-seeking. For example, if one where to recognise the problem as either depression or anxiety, where anxiety is often perceived by the general public as a less severe problem and generally perceived as having less need for treatment [40], the individual may decide to not seek professional help. These findings highlight the need to build on Sri Lankan Australians’ knowledge of depression specifically in order to differentiate its symptoms from other conditions [40]. Perhaps the most important aspect to note for future studies pertains to the use of the vignette methodology to identify MHL. In line with the considerable critical literature on vignette methodology [45,46], this study showed how a vignette illustrating depression consistent with DSM-5 diagnostic criteria could yield multiple labels, including more normalizing language such as stress and burnout. However, it has been argued that these alternative labels are less likely to facilitate professional help seeking [17]. The research team recommends that future research pay attention to different operationalisations of the “correct” recognition of depression when using this methodology and directly test how the use of multiple labels may influence future treatment seeking.

### 5.1. Predictors of Recognition of Depression

Other findings from the study offered more specific directions for mental health promotion and interventions. Inconsistent with findings from Chinese subjects in Australia [31], in the current study, acculturation was not an independent predictor of recognising depression. The results demonstrated that although acculturation uniquely predicted one’s ability to recognise depression, once other demographic and exposure to depression variables were controlled for, the relationship between acculturation and problem recognition decreased. Perhaps these findings highlight the clinical significance of conceptualising Sri Lankan Australians as multidimensional rather than perceiving them as belonging to only one social group [47]. Utilising an intersectionality framework when working with this population would remind mental health clinicians that they cannot understand Sri Lankan Australians’ experience of one social group membership (e.g., Australian identified) without reference to their other social group memberships (e.g., age, education, personal experience of mental health), and that the interactive role of experiences might influence their mental health and help-seeking behaviours.

As predicted, the finding that younger Sri Lankan Australians have higher recognition of depression than their older counterparts aligns with previous problem recognition studies among the general Australian population [24]. While such findings might reflect actual MHL deficits among older Sri Lankans in Australia, they might also be explained by this group holding the belief that depression is “normal” for older adults [30]. The low level of correct identification of depression in this subpopulation points to the need to improve knowledge of disorder symptoms among older migrant communities, especially as help-seeking is often mediated by family members [28] in collective and familial orientated communities. In fact, research has consistently demonstrated that individuals in these societies do not make decisions solely on their own, but rather consider the views of informal sources, especially elders, before seeking professional help [3,18].

As would be expected, participant’s past diagnosis of depression was predictive of their ability to recognise depression. This finding concurred with previous findings [31] and could be indicative of increased encounters with psychoeducation [27].

### 5.2. Limitations

The present study had several limitations that should be noted. First, recruitment into the survey was originally planned to be via two channels: social media and face-to-face (particularly through networks developed with key community organisations). The COVID-19 pandemic made the latter channel unfeasible, and consequently the final sample was skewed towards the younger population recruited through social media. Older Sri Lankan Australians were both scientifically and clinically significant and were hypothesised to have significantly different levels of recognition compared to younger, more acculturated Sri Lankan Australians. Thus, a major limitation of the overall sample was the fact that less than 10% of participants were over 50 years of age. Therefore, it would be desirable to replicate these findings with a more diverse age group, and generalisations to the Sri Lankan Australian population as a whole must be made with caution.

Second, due to having limited cases within some sub-categories of the ethnicity, country of birth and religion variables, the research team decided to collapse the aforementioned categorical predictor variables. Subsequently, some interesting and nuanced data about the Moor, Burgher, Chinese Sri Lankan and Malay populations may have been removed. Additionally, the disproportionate underrepresentation of Tamil participants and the overrepresentation of Sinhalese participants were significant weaknesses of the current study and limited the interpretation and generalisation of the results. Future studies should prioritise recruiting from the range of ethnic and religious groups that make up the Sri Lankan diaspora in Australia in order to deepen the MHL knowledge base and inform future health promotion efforts.

Third, the research team is aware of the limitations of only distributing the survey in English. Ideally, the survey would have been also distributed in Sinhala and Tamil; however, this was unfeasible due to limited resources. Lastly, despite MHL’s established utility as a structure for understanding factors that influence individuals’ mental health help-seeking [48,49], many concerns exist regarding the concept’s ecological validity [20], vignette-based methodology [50], focus on individual diagnosis [48] and lack of acknowledgment of potential variations in the expression of mental health conditions across cultures [48]. Nonetheless, as this was the first survey investigating MHL in Sri Lankan Australians, we believed it was advantageous to employ a concept and methodology that has been used in over five hundred papers [20] including with other Sri Lankan populations [18] and Asian migrant populations in Australia [3]. Future research could use other measurements of MHL that address the limitations of vignette-based methodologies.

## 6. Conclusions

One’s ability to accurately recognise depression is a factor that can facilitate appropriate help-seeking behaviours and, in turn, access to effective treatment [51]. The results demonstrated that Sri Lankan Australians’ ability to recognise depression was comparable to the general Australian population. As expected, Sri Lankan Australians’ own experience with depression and younger age were predictive of their ability to recognise depression. These findings could guide future MHL interventions aimed at increasing access to mental health services. For example, future interventions could target specific subpopulations which appear to be less likely to recognise depression, such as older Sri Lankan Australians or individuals with limited experience with mental health conditions and treatment. Given the relatively high recognition rate of depression, the current findings also suggested that there may be other factors that act as barriers to help-seeking among the Sri Lankan Australian community. For example, pragmatic barriers, stigma and access to culturally responsive services including translation services and bilingual mental health professionals may influence help-seeking and need to be further investigated in future research in order to deepen our understanding of the mental health experiences of the Sri Lankan Australian population.

## Figures and Tables

**Table 1:** Demographic and other characteristics of the sample (*N* = 404).

Variables	Frequency	%
**Gender**		
Male	120	29.7
Female	284	70.3
**Age group (*Mean = 31.6; SD = 11.6*)**		
18–29 years	237	58.7
30–39 years	94	23.3
40–49 years	34	8.4
50–59 years	24	5.9
60–69 years	11	2.7
70 and over	4	1.0
**Highest level of education**		
Secondary school or equivalent	50	12.4
TAFE course or equivalent	32	7.9
Undergraduate degree	190	47.0
Postgraduate degree	132	32.7
**Country of birth**		
Australia	127	31.4
Sri Lanka	252	62.4
Other	25	6.2
**Years in Australia (*Mean* = 17.8; *SD* = 10.0)**		
Under 10 years	110	27.2
11 to 20 years	101	25.0
21 to 30 years	165	40.8
Above 30 years	28	6.9
**Ethnicity**		
Sinhalese	343	84.9
Tamil	27	6.7
Other	34	8.4
**Religion**		
Buddhist	247	61.1
Christian	29	7.2
Roman Catholic	49	12.1
Atheist or agnostic	43	10.6
Other	36	8.9

Note. Standard deviation (*SD*), technical and further education (TAFE).

**Table 2:** Problem recognition (*N* = 404).

Label Category	Percentage Recognised (95% CIs) (*N* = 404) ^1^
Depression recognised	73.8 (69.3–78.2)
Depression unambiguously recognised	53.7 (49.0–58.9)
Anxiety	14.9 (11.1–18.6)
Stress/mental suffering	8.4 (5.7–11.1)
Mental problem	6.4 (4.2–8.7)
Mental illness ^2^	4.7 (2.7–6.7)
Emotional symptoms	4.2 (2.2–5.9)
Has a problem (neither physical nor mental)	3.0 (1.5–5.0)
Social withdrawal and isolation	1.7 (0.5–3.2)
Physical condition	5.0 (3.0–7.2)
Lack of motivation	2.0 (0.7–3.7)
Adjustment	3.0 (1.5–4.7)
Burnout	1.7 (0.5–3.2)
Lack of communication	1.7 (0.7–3.0)
Trauma/loss	1.0 (0.2–2.2)
Don’t know	1.2 (0.2–2.5)
Other	1.7 (0.7–3.2)

Note. Confidence intervals (CIs). % Sample indicates use of the label categories in relation to the total sample. ^1^ Apart from the “don’t know” category, the pattern of findings did not differ when assessing the subset of the sample (262 participants) who completed the full survey. ^2^ The “mental illness” category constituted a separate category that included all other diagnoseable mental illnesses that were not captured in the depression or anxiety categories.

**Table 3:** Predictors of recognition of depression examined using sequential binary logistic regression (*n* = 262).

Model	Predictor	B	SE	Wald	*p*	Odds Ratio	95% CI for Odds Ratio		
							Lower	Upper	Percent Variance Explained/Chi-square Change
Model 1	Acculturation	0.88	0.23	14.29	0.00	2.40	1.52	3.78	8.5/15.26
	Constant	−1.43	0.68	4.37	0.04	0.24			
Model 2	Acculturation	0.30	0.27	1.24	0.27	1.35	0.80	2.29	19.7/21.50
	Age	−0.07	0.02	19.40	0.00	0.94	0.91	0.97	
	Constant	2.45	1.13	4.68	0.03	11.60			
Model 3	Acculturation	0.24	0.28	0.74	0.39	1.27	0.74	2.19	22.5/5.72
	Age	−0.07	0.02	18.80	0.00	0.94	0.91	0.97	
	Diagnosis of depression	0.91	0.40	5.15	0.02	2.48	1.13	5.44	
	Constant	2.42	1.16	4.33	0.04	11.25			
Model 4	Acculturation	0.10	0.28	0.10	0.75	1.10	0.63	1.90	26.3/7.98
	Age	−0.05	0.02	11.26	0.00	0.95	0.92	0.98	
	Diagnosis of depression	1.07	0.42	6.51	0.01	2.93	1.28	6.67	
	**Highest level of education**								
	TAFE course or equivalent	−2.11	0.93	5.11	0.02	0.12	0.02	0.76	
	Undergraduate degree	−0.81	0.79	1.06	0.30	0.44	0.10	2.08	
	Postgraduate degree	−1.42	0.81	3.12	0.08	0.24	0.05	1.17	
	Constant	3.55	1.37	6.70	0.01	34.73			

Note. *B* coefficient (B), standard error (SE), Wald test (Wald), *p*-value (*p*), confidence interval (CI), technical and further education (TAFE). Reference categories: secondary school or equivalent; no diagnosis of depression.

## Data Availability

The dataset presented in this article is not publicly available, but the dataset is available from the senior author on reasonable request. Requests to access the dataset should be directed to Greg Murray, gwm@swin.edu.au.
